# Insight orientation scale: A promising tool for organizational outcomes–A psychometric analysis using item response theory

**DOI:** 10.3389/fpsyg.2022.987931

**Published:** 2022-09-02

**Authors:** Alessio Gori, Eleonora Topino, Andrea Svicher, David Schuldberg, Annamaria Di Fabio

**Affiliations:** ^1^Department of Health Sciences, University of Florence, Firenze, Italy; ^2^Department of Human Sciences, LUMSA University of Rome, Rome, Italy; ^3^Department of Education, Languages, Intercultures, Literatures and Psychology (Psychology Section), University of Florence, Firenze, Italy; ^4^Department of Psychology, The University of Montana, Missoula, MT, United States

**Keywords:** item response theory, insight, self-report scale, assessment, insight orientation scale, IOS

## Abstract

Insight is a construct carried out into different theoretical orientations with increasing application out of the boundaries of clinical psychology. Recent studies have investigated insight also as a promising variable for organizational outcomes. Given the relevance of Insight in promoting change, this paper aimed at describing the psychometric analysis of one of the shortest, most agile, and most versatile tool for measuring some of the characteristics of insight, the Insight Orientation Scale (IOS), using Item Response Theory. To achieve this goal, we applied a Mixed Rash Model to the IOS. Data from 1,445 individuals were analyzed by the means of WIN-MIRA and Multilog. Based on the likelihood statistics (CAIC) we assumed a three-class solution for the IOS. Results also indicated that the greater part of items had good discrimination and threshold parameters. These findings confirmed psychometric stability of the IOS highlighting its measurement precision, supporting its utility in both research and practice.

## Introduction

In the APA *Dictionary of Psychology* insight is defined as “the clear and often sudden discernment of a solution to a problem by means that are not obvious and may never become so, even after one has tried hard to work out how one has arrived at the solution” (VandenBos, 2007, p. 484); in other terms, insight can be conceptualized as a conscious change of meaning that implies new connections (Hill et al., 2007) and as a part of the creative process. Insight was initially applied as a construct within the psychotherapy treatment (e.g., Timulak and McElvaney, 2013) linked with favorable outcomes (e.g., Hill et al., 2007; Gibbons et al., 2009; Gabbard, 2014; Lacewing, 2014). Because this construct has gained a key role in psychotherapy treatment it is therefore the object of growing clinical and research attention. The acquisition of insight favors a greater awareness of the defense mechanisms of their role in the patient’s life (Rosenblatt, 2004; Gabbard, 2014), the link between past experiences and actual psychological issues (Hill et al., 2007), and distorted perceptions of self and others (Lacewing, 2014). Achieving insight is linked with increasing the ability to master symptoms (Rosenblatt, 2004), develop more adaptive behaviors (Gabbard, 2014), and improve quality of life (Gibbons et al., 2009). However, in recent years, scholars have expanded insight research beyond the boundaries of the clinical domain (Gori et al., 2021a). In this framework, researchers have highlighted the promising role of insight in the realm of Work and Organizational Psychology (Gori et al., 2021b). Recent empirical evidence grounded in the Work and Organizational Psychology field are encouraging the study of insight as a variable related to workers and work-related outcomes (e.g., Gori and Topino, 2020). Results from the workplaces showed that insight orientation mediates the relationship between the Big Five personality traits and job crafting (Gori et al., 2021b). More particularly, insight orientation mediates the association between extraversion and job crafting, suggesting that insight could foster workers’ assertiveness/energy, thereby leading them to better craft their jobs (Gori et al., 2021a), being also promising for vulnerable workers (Svicher and Di Fabio, 2021). Research has also shown that insight orientation together with workplace rational civility mediates the link between predisposition to change and job satisfaction, thus contributing to positive and mutually supportive links among coworkers (Gori and Topino, 2020). Furthermore, Gori et al. (2021b) reported that insight in workers also mediates the relationship between trait emotional intelligence and acceptance of change, highlighting the role of insight as a promising primary preventive resource for promoting acceptance of change in organizations (Gori et al., 2021b).

Starting from these premises, insight seems to be a promising construct also for applied research in Work and Organizational Psychology. In this regard, there are several approaches to operationalizing the construct of insight, some of them are narrowed in what follows to awareness of mental disorder while others are more interestingly related to the description and elaboration of subjective feelings, sensations, and reactions.

### Assessment of insight

Although the concept of insight has gained a lot of attention over the years, until recently there has been a lack of standardized assessment tools for insight. One of the first developed and widely used tool was the Insight and Treatment Attitudes Questionnaire (ITAQ) advanced by McEvoy et al. (1981). The ITAQ is a questionnaire with 11 items that assess participants’ attitudes about whether they have a mental disorder and whether they need treatment. Responses provided by subjects are scored from good insight (2) to no insight (0) with two overall scores, namely awareness of possessing mental disorders and awareness of need for intervention. Although predictive of a wide array of outcomes and compliance (McEvoy et al., 1981), this measure lacks the assessment of domains that other scholars believe comprise insight, mainly continuing the tradition of conceptualizing insight as a unitary phenomenon strictly related to awareness of mental disorders. Differently, Amador et al. (1991, 1993); Amador and Strauss (1993); and David (1990) have adopted a multidimensional approach to insight as conceived by distinct but overlapping factors. Amador et al. (1991, 1993) distinguished two dimensions of insight (i.e., awareness of illness and attribution regarding illness), whereas David (1990) identified three dimensions (recognition of having a mental disorder; compliance with treatment; and capacity to label unusual events as pathological, for example, delusions and hallucinations).

In addition, Birchwood et al. (1994) developed the Insight Scale for Psychosis, a self-report measure that parallels the three dimensions of Davis but with a specific focus on change in insight during time. Accordingly with this concept of insight, Amador and Strauss (1990) purposed the Scale to assess Unawareness and Mental Disorder (SUMD) hat adds three dimensions focused on current and retrospective awareness of: (1) having a mental disorder; (2) the effects of medications; (3) the consequences of mental disorder; (4) and specific signs and symptoms. The SUMD evaluates subjects’ endorsement of a diagnostic label *via* 74 items rated on five-point Likert scale, however, it does not measure belief associated with the need of a treatment, which can be a major limitation of the scale (Amador and David, 2004).

Other approaches have included using items of the Positive and Negative Syndrome Scale (PANSS; Kay et al., 1987) and of the Present State Examination (PSE; Wing et al., 1974) as single global measures of insight. The Positive and Negative Syndrome Scale (PANSS; Kay et al., 1987) has one single item measuring insight enclosed in the General Psychopathology subscale (item G12). The subject is assessed on a scale from 1 (“absence or lack of judgment and insight”) to 7 (“extreme lack of judgment and insight.”). The Present State Examination (PSE; Wing et al., 1974) also has a single insight item (the total number of items is 104) that rates responses to probe questions (“Do you think there is anything the matter with you?”) on a scale from 0 to 3. However, both PANASS and PSE were found to be not able to measure nuances of insight. Moreover, their reliance on only one item makes them with weak psychometric properties (Amador and David, 2004).

Most of the measures evaluate insight during psychotherapy, such as the Private Self-Consciousness Scale (PrSCS; Fenigstein et al., 1975) or the Self-Reflection and Insight Scale (SRIS; Grant et al., 2002), describe stages of elaboration of thoughts and ideas that vary from avoidance to consciousness, from superficial comprehension to insight and elaboration, to change of meaning, resolution of problem or integration (Klein et al., 1986; Stiles et al., 1990; Angus et al., 1999; Greenberg, 2002). The PrSCS analyzes two factors, named Self-Reflection and Internal State Awareness, and the SRIS as well is composed of two different dimensions named Self-Reflection (SRIS-SR) and Insight (SRIS-IN) (Grant et al., 2002).

Other scales, such as the Experiencing Scale (Klein et al., 1986), the Assimilation of Problematic Experiences Scale (APES) (Stiles et al., 1990), and the Narrative Processes Coding System (NPCS) (Angus et al., 1999) are well known in the research as measures of aspects of insight related to experience. The Experiencing Scale (Klein et al., 1986) measures the involvement of clients in terms of principal aspects in counseling not only in psychotherapy. For Klein et al. (1986) the clients’ experience is related to their attention. The scale has a hierarchical structure measuring client statements taking into account feelings about internal, impersonal, and external referents (Ulak and Cummings, 1997).

The APES is a tool that measures the assimilation process through eight-stages rated along a continuum from experiences that are avoided or averted from experiences that are understood, integrated, and resolved in the self. The sequence is summarized in the eight stages or levels of the Assimilation of Problematic Experiences Scale (APES), numbered 0 to 7: (0) Warded off/dissociated; (1) Unwanted thoughts/active avoidance; (2) Vague awareness/emergence; (3) Problem statement/clarification; (4) Understanding/insight; (5) Application/working through; (6) Resourcefulness/problem solution; and (7) Integration/mastery. The APES considers both affective and cognitive aspects encompassed in all the eight levels. However, these features are rated using anchor points in a continuum instead of discrete indicators.

The NPCS is a systematized methodology for analyzing session transcripts (Angus et al., 1999). According to this system, three distinct narrative process modes can be discerned during sessions. External narrative entails pictures of events; internal narrative deals with describing and elaborating subjective sensations, feelings, and reactions; reflexive narrative encompasses features associated with individuals’ meaning. The NPCS allows the researcher to track shifts both in topics and types of processes involved in narratives encased in a client’s conversation, within and across sessions. This, in turn, allows comparisons between different groups, outcome and interventions (Angus et al., 1999).

Other important tools applied to evaluate features of insight are the Change and Growth Experiences Scale (CHANGE; Hayes et al., 2007a) and the Beck Cognitive Insight Scale (BCIS; Beck et al., 2004). The CHANGE is a coding system to measure clients’ processes as intervention progresses. The coding system encompasses the assessment of psychological functioning of a client in various domains (interpersonal, affective, cognitive, behavioral, and somatic), as well as avoidance and processing in the cognitive-emotional domain. Each variable is coded on a scale from 0 (not present or very low) to 3 (high) (Hayes et al., 2007b). The BCIS is a self-administered scale composed of 15 items ranked on a 4-point Likert scale from 0 (do not agree at all) to 3 (agree completely) *via* two factors: (1) *Self-Reflectiveness*; and (2) *Self-Certainty*.

On the one hand, this brief review of measures for assessing insight testifies the attention of researchers to phenomena related to a multi-facetted construct of insight and, on the other hand, it tries to underline some limitations with the construct and its measurement.

### The insight orientation scale

Recently, Gori et al. (2015) developed the Insight Orientation Scale (IOS), a brief and agile self-report instrument measuring insight as a conscious element (Hill et al., 2007): “a conscious meaning shift involving new connections” (p. 442). The scale has shown good indications of internal consistency, as well as concurrent and discriminant validity in subjects with the different diagnosis, and, given its brevity and agility of administration and scoring (7 items on the Likert scale), the IOS is particularly attractive both in applied research and practice (Prieto et al., 2003). Parallelly, the scale focuses specifically on insight orientation with a solid and comprehensive theoretical basis, structuring itself with items that explore and investigate 7 central elements of the construct (Castonguay and Hill, 2007; Hill et al., 2007): surprise, restructuring, level of consciousness, problem solving, complexity, self-reflectiveness, and awareness (Gori et al., 2015). In light of these properties, the IOS appears to be a particularly functional scale and has been used to facilitate the understanding of processes not only in the clinical context (Gori et al., 2022), but also in different applied contexts as the workplace (Di Fabio and Gori, 2016a; Gori and Topino, 2020).

### The present study

Since Insight Orientation Scale (IOS; Gori et al., 2015) is a short, agile, theoretically grounded, jargon-free scale, promising for use in a variety of practice and research contexts. Thus, it appears functional to deepen its statistical solidity by testing its goodness based on recent advances in psychometric theory. Therefore, the aim of this study is to detail the psychometric properties of IOS (Gori et al., 2015) through the practice of item response theory (IRT), to enrich and implement the knowledge of the characteristics of this measure and encourage its conscious use not only in the clinical but also in the work and organizational context.

## Materials and methods

### Participants and procedure

Data from 1,445 individuals (39.2% male, 60.8% female) were analyzed *via* the Windows Mixed Item Response Analysis (WIN-MIRA; von Davier, 2001a) and the Multilog software (Multilog 7.0.3; Thissien, 2003). All participants, with ages ranging from 18 to 85 years (*M* = 31.8; SD = 12.18; Male *M* = 33.73, *SD* = 13.21; Female *M* = 31.42, *SD* = 11.54), were Italian and completed the Insight Orientation Scale (IOS; Gori et al., 2015) in booklet form. Participants were recruited both from the university and from the general community; they were informed about the aim of the study and agreed to collaborate. The time for administration ranged from approximately 5–10 min for each questionnaire.

### Measure

The *Insight Orientation Scale* (IOS; Gori et al., 2015) is a 7-item self-report scale used to assess the orientation and tendency toward insight, by exploring some specific characteristics that could help in this process (surprise, restructuring, level of consciousness, problem solving, complexity, self-reflectiveness, and awareness). Items (e.g., “I am able to be reflective about myself”) are rated on a five-point Likert scale, (from 1 = “not at all” to 5 = “a great deal”). The scale provides for the possibility of calculating a total score ranging from 7 to 35, such that higher scores on the IOS indicate higher levels of insight.

### Data analysis

In order to investigate the distribution of the data in participants, descriptive statistics were calculated. The Rash properties of the IOS were investigated using the Mixed Rasch Model, which extends the Rasch model to a discrete mixture model. This model allows researchers to analyze the items of scales with ordinal response formats to detect possible inhomogeneous samples into Rasch homogenous subsamples and provide profile analysis of responses. Using the WINMIRA (von Davier, 2001a,b) specifically developed to calculates polytomous (ordinal) Rasch models, two different models were evaluated to find the model with the maximum fit to our data: the Rating Scale Model (RSM; Andrich, 1978) and the Partial Credit Model (PCM; Masters, 1982).

In the RSM, each item of IOS has the same number of thresholds, and threshold locations and the mean of the threshold locations is equal or uniform across all the items. In the PCM each item of IOS has one parameter and does not have restrictions with the exception of the common constraint of having a normalizing condition. The PCM is similar to the conventional Rasch model (Rasch, 1960) but allows items with an ordered categorical response format, whereas the conventional Rasch model assumes only dichotomous responses. In The PCM model, whether a person parameter or latent trait (q) reaches a particular amount that is behind a certain level, a response will most likely fall into a specific category (Erhart et al., 2009).

In line with Sober (2002), the Akaike Information Criterion (AIC) permits researchers to trustworthy calculate the best model considering a parsimony principle grounded on observable evidence. The AIC has two elements: the deviance (*d*), calculated *via* posterior means ability parameters of items, and 2 * *p* (number of estimated parameters), interpreted as a penalty function for over-parameterization (Sung and Kang, 2006). Thus, the AIC is defined as:


(1) AIC⁢(Model)=d+2⁢p

According to this model, The Model with the smallest AIC has the best fit to the data.

With the Mixed Rasch Model it is possible to identify homogeneous subpopulations in a heterogeneous sample. Within these latent classes the item parameters are the same for each individual, and the subject’s scores take meaning according to the belonged-to class. Between the classes there are qualitative individual differences, not only quantitative differences. To find the best fit of an underlying trait, the Consistent Akaike Information Criterion (CAIC) (Bozdogan, 1987) was implemented. The CAIC’s equation is:


(2) CAIC=-2logL+N[1+log(N)]par

The *N* is referred to the observed sample size, *N*_*par*_ indicates the number of parameters in the model and *log (N)* represents the log-likelihood of the model. According to this latter criterion, the model that shows the lowest CAIC has the best fit to data. In the realm of Mixed Rasch Model, the Windows-Mixed Item Response Analysis software (WIN-MIRA; von Davier, 2001a), has the possibility to estimate also the parameters of the Rasch Model for each homogeneous subpopulation. The Mixed Rasch Model calculates for each item of a scale the difficulty parameter and the threshold parameters. Given that the IOS items are ranked on a 5-point Likert scale, they have four item threshold parameters because *J* = *K – 1* (where *K* is the number of category responses). In addition, since the Mixed Rasch Model is a probabilistic mode, the maximum likelihood can be calculated using two procedures, the Maximum Likelihood Estimation (MLE) and Weighted Likelihood Estimation (WLE). WLE shows two main advantages as compared to the MLE because it has smaller bias and it produces reasonable estimates also in the case for responses with extreme patterns i.e., for the patterns with zero and the maximum observable score (Warm, 1989; Hoijtink and Boomsma, 1995). Furthermore, to overcome issues related with an estimating biased person and item parameters simultaneously the WIN-MIRA also used the Conditional Maximum Likelihood Estimation (CML).

Item fit was assessed *via* the Q-index (Rost and von Davier, 1994) which compares the likelihood *p*(*X*obs) of the empirical item response vector to the maximum *p*(*X*max) and minimum likelihood *p*(*X*min) which it could theoretically achieve.


(3) $$Q=\frac{\text{log}\,\left[p\,\left(\underline{X}\,\mathrm{obs}\right)/p\,\left(\underline{X}\,\max\right)\right]}{\text{log}\,\left[p\,\left(\underline{X}\,\min\right)/p\,\left(\underline{X}\,\max\right)\right]}$$

Q-values above 0.30, or a statistically significant deviation of the empirical Q-index from its expected value, indicates a misfit of one item. This means that the empirical responses to the item cannot be satisfactorily predicted using the model (Erhart et al., 2009).

In addition, to take a complementary IRT approach for analyzing the psychometric properties of the IOS, an additional IRT analysis was also conducted using Multilog 7.0.3 (Thissien, 2003). As the IOS (Gori et al., 2015) has a polytomous response format, the Graded Response Model (GRM; Samejima, 1969) represents a potentially appropriate IRT model that has been successfully applied with a few polytomous personality-type measures (e.g., Gomez et al., 2005; Rubio et al., 2007; Cooper and Petrides, 2010); it has been considered one of the most often applied model for use with interval scale data (Lautenschlager et al., 2006). With the Graded Response Model, Item Characteristic Curves (ICCs) are generated. ICCs illustrate the likelihood of endorsing a response category taking into account the different levels of the underlying latent trait (e.g., Insight) necessary to endorse the examined response (Cooper and Petrides, 2010). The GRM provides two item parameters. The first is Difficulty (b), and the second is discrimination parameters (*a*). Discrimination parameters in the GRM (*a*) constrained equal for the response categories within an item but are free to vary across the different items. The GRM also provides an information function for a global test, called Test Information Function (TIF). This function shows a curve that illustrates the precision of the measurement of the test among different levels (theta) of the latent trait. TIF also shows the Standard Errors of Measurement (SEM) of the functions that indicate the precision of a test at the different levels of the measured latent trait (Embretson and Reise, 2000). In terms of model-data fit Multilog provides an estimate for each response option considering the observed and expected proportion of responses. In this vein, the expected proportion and estimated values are based on the item parameters and latent trait, and size of the residuals can provide information on model-data fit, with greater residuals suggesting poorer fit (e.g., Cooper and Petrides, 2010).

## Results

### Descriptive statistics

To investigate the differences on levels of insight between subpopulations we calculated the IOS total score, separately for men and women and for five age groups (18–23; 24–35; 36–45; 46–55; 56–65; 65 years old and over). An independent samples *t* test indicated that there were not significant differences between men and women on IOS scores (*t* = 0.39, *p* < 0.47). An ANOVA showed that there were no differences related to age groups on the IOS scores (*F* = 0.15, *p* = 0.69).

### Item response theory analysis: WINMIRA

Choice of the model and number of latent classes was determined by first checking fit indices, in particular the AIC and CAIC. In order to choose the best-fitting model we compared the Akaike (AIC) indices obtained with the PCM and the RSM methods and selected the Partial Credit Model because it yielded the smallest AIC’s values (see Table 1).

**TABLE 1:** Information criteria: The AIC and CAIC values for the PCM and RSM.

Class	Index	Partial Credit Model (PCM)	Rating Scale Model (RSM)
1	AIC	24,853.66	25,483.17
	CAIC	25,035.32	25,552.13
2	AIC	24,574.76	24,805.20
	CAIC	24,615.14	24,949.38
**3**	**AIC**	**24,026.52**	24,582.48
	**CAIC**	**24,584.02**	24,801.89
4	AIC	24,482.14	24,626.15
	CAIC	24,737.38	24,920.79

Values in bold indicate the best-fitting solution.

Latent class models with one to four classes were used to select the best-fitting solution. Evaluating all of the models according to the CAIC-values, the three class model was chosen as the best-fitting solution for the data; this means that there are three subsamples that respond differently to the items, probably because they have different levels of the underlying trait. The CAIC value is always higher in class 1, in class 2 and, in class 4, than in the 3 class model (see Table 1).

**FIGURE 1:**
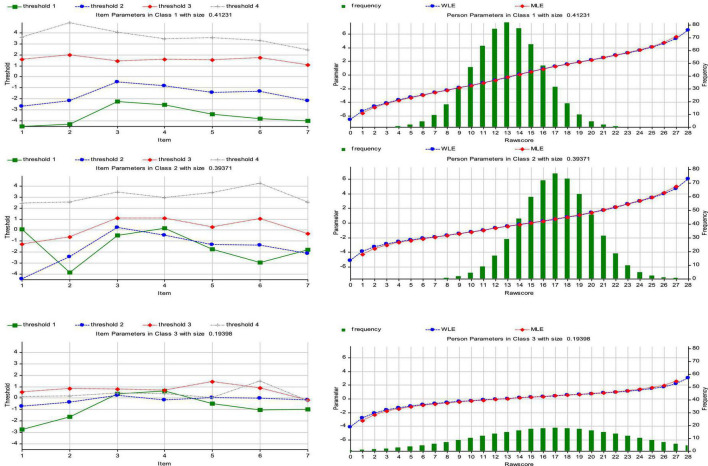
Item Parameters and Person Parameters – Partial Credit Model. The Item Parameter graph shows the threshold for each one of the seven items of the IOS. The Person Parameter graph show the absolute raw score frequencies for each one of the three class solutions.

The Item Parameters and the Person Parameters of the three classes are shown in Figure 1 (see Figure 1).

According to Rasch PCM, threshold parameters of items have to be ordered; each parameter should change from threshold to threshold since an ordered response format is assumed. An appropriate change of the threshold parameters reflects that each response category is representative for an ordered interval of the individual parameter dimension. In Latent Class 1 (LC1), the class size shows that around 41% of the participants can fit under a polychotomous Rash model which was assumed to hold in this class. In this subsample the thresholds for each item are more or less equidistant and follow a homogeneous trend: for example, as regards the item 1, the values range from −4.52 to 3.62 (threshold 1 = −4.52; threshold 2 = −2.72; threshold 3 = 1.57; threshold 4 = 3.62) (see Table 2). In addition, Table 2 shows the values of item location that represent overall difficulty parameters. Items with low (or negative) values indicate relative easiness for examinees in choosing the correct response, as compared to high (or positive) values which indicate more difficulty in selecting the correct (keyed) response; in the case of a trait measure, “difficulty” corresponds to the item being readily endorsed even by participants relatively low on the actual trait. Each item showed different ease of endorsement levels for subjects in distinct latent classes. For instance, Item 2 had the location parameters of 0.085 (see Table 2), −1.103 (see Table 4), and−0.269 (see Table 5) for Latent Classes 1 (LC1), 2 (LC2), and 3 (LC3), respectively. That is, subjects classified in Latent Class 2 found it easier to answer in the keyed direction than did subjects in Classes 1 and 3. Examination of the item-fitting indices showed a satisfactory fit of the items to the data (*Q* = 0.14–0.22) (see Table 2).

**TABLE 2:** Item threshold parameters and item fits assessed by the Q-index – Partial Credit Model.

Threshold parameters: ordinal (partial credit) model						Item fit assessed by the Q-index		
	
Item Label	Item Location	threshold parameters				Q-index	Zq	*p*(X > Zq)
					
		1	2	3	4			
Item1	−0.51183	−4.522	−2.72	1.574	3.621	0.2248	0.4135	0.33963
Item2	0.08501	−4.319	−2.218	1.966	4.911	0.1633	−0.466	0.67938
Item3	0.6773	−2.272	−0.507	1.449	4.039	0.1477	0.0503	0.47996
Item4	0.41685	−2.539	−0.817	1.593	3.43	0.1363	−0.6513	0.74256
Item5	0.05151	−3.408	−1.468	1.527	3.555	0.1957	0.4083	0.34153
Item6	−0.04233	−3.835	−1.367	1.725	3.309	0.1754	−0.106	0.54222
Item7	−0.67651	−4.003	−2.185	1.057	2.426	0.1825	0.2806	0.38952

Class 1 of 3 with size 0.41231.

The frequency distribution of the raw scores of subjects in the three latent classes and probabilities of answering items in the keyed direction or not can provide further evidence to support the descriptions of the three latent classes found in this study. For Person Parameters, both MLE and WLE are shown. The Person Parameters graphs show the absolute raw score frequencies for each one of the three classes (every class was assumed to be Rasch homogenous), along with a simultaneous person parameter plot for the Maximum Likelihood and the Warm Person Parameter (see Figure 1). The values of LC1 are shown in Table 3 (see Table 3).

**TABLE 3:** Person Parameters: CLASS 1 of 3 with size 0.41231 – Partial Credit Model.

Score frequency		Person parameters and standard errors:			
	
Raw – Score	Expected freq.	MLE – Estimate	Std. Error MLE	WLE – Estimate	Std. Error WLE
0	0	**** ****	**** ****	−6.527	1.554
1	0.01	−5.625	1.073	−5.282	0.949
2	0.05	−4.776	0.813	−4.615	0.779
3	0.18	−4.206	0.709	−4.112	0.696
4	0.58	−3.743	0.655	−3.685	0.65
5	1.63	−3.336	0.624	−3.3	0.622
6	4.06	−2.959	0.606	−2.938	0.605
7	8.98	−2.598	0.597	−2.589	0.596
8	17.58	−2.245	0.594	−2.246	0.594
9	30.48	−1.89	0.598	−1.9	0.598
10	46.81	−1.527	0.607	−1.546	0.606
11	63.67	−1.151	0.62	−1.174	0.619
12	76.7	−0.759	0.634	−0.779	0.633
13	81.84	−0.351	0.643	−0.358	0.643
14	77.34	0.063	0.642	0.075	0.641
15	64.73	0.468	0.629	0.495	0.628
16	47.98	0.852	0.611	0.883	0.609
17	31.5	1.214	0.593	1.24	0.592
18	18.32	1.558	0.58	1.574	0.58
19	9.43	1.891	0.574	1.896	0.574
20	4.3	2.219	0.574	2.213	0.574
21	1.74	2.552	0.581	2.535	0.581
22	0.62	2.898	0.596	2.868	0.594
23	0.2	3.267	0.62	3.221	0.617
24	0.06	3.673	0.658	3.605	0.651
25	0.01	4.143	0.719	4.041	0.704
26	0	4.733	0.829	4.567	0.793
27	0	5.616	1.092	5.275	0.973
28	0	**** ****	**** ****	6.581	1.595

WLE, warm’s modified likelihood estimates; MLE, standard maximum likelihood estimates; ******, MLE estimates for extreme score groups are not provided by WINMIRA, because for extreme scores, the class-specific expected frequencies cannot be compared to the observed frequencies using MLE. For extreme scores, only WLE estimates are provided by WINMIRA since they are less biased and give more reasonable estimates (von Davier, 2001b).

**TABLE 4:** Item threshold parameters and item fits assessed by the Q-index – Partial Credit Model.

Threshold parameters: ordinal (partial credit) model						Item fit assessed by the Q-index		
	
Item Label	Item Location	Threshold parameters				Q – index	Zq	*p* (X > Zq)
					
		1	2	3	4			
Item1	–0.8192	0.072	–4.466	–1.31	2.427	0.2773	–0.0909	0.53619
Item2	–1.1037	–3.876	–2.434	–0.642	2.536	0.2442	–0.0718	0.52861
Item3	1.06443	–0.48	0.218	1.063	3.457	0.1369	–0.4713	0.68128
Item4	0.91829	0.16	–0.508	1.093	2.928	0.1451	–0.1123	0.54472
Item5	0.15128	–1.729	–1.332	0.288	3.377	0.2369	0.3872	0.34932
Item6	0.22584	–2.961	–1.418	1.035	4.247	0.2774	0.6776	0.24902
Item7	–0.4369	–1.802	–2.146	–0.33	2.53	0.2269	–0.2884	0.61347

Class 2 of 3 with size 0.39371.

**TABLE 5:** Item threshold parameters and item fits assessed by the Q-index – Partial Credit Model.

Threshold parameters: ordinal (partial credit) model						Item fit assessed by the Q-index		
	
Item Label	Item Location	Threshold parameters				Q – index	Zq	*p* (X > Zq)
					
		1	2	3	4			
Item1	–0.71154	–2.771	–0.725	0.537	0.113	0.1212	–0.6674	0.74775
Item2	–0.2693	–1.669	–0.394	0.823	0.163	0.1191	–0.5912	0.72279
Item3	0.44203	0.377	0.205	0.766	0.420	0.1565	0.8945	0.18554
Item4	0.37301	0.613	–0.175	0.686	0.368	0.1239	–0.0283	0.51128
Item5	0.23893	–0.472	0.003	1.420	0.004	0.1330	0.0384	0.48469
Item6	0.32483	–1.028	–0.011	0.881	1.458	0.1756	0.4572	0.32375
Item7	–0.398	–0.975	–0.185	–0.207	–0.226	0.1420	–0.0209	0.50834

Class 3 of 3 with size 0.19398.

Table 3 shows the expected raw score frequencies concerning Class 1, the Person parameter estimate and the standard error estimation of the individual parameter for all raw scores in class one. The raw-score mean was 13.034 (SD = 2.87).

With regard to the threshold parameters in Class 2 with size 0.39371, the trend is almost the same of the corresponding parameter in class 1, except for the first thresholds of item 1 (0.072), item 4 (0.160) and item 7 (−1.802) (see Table 4). For instance, in the case of item 1 in LC2, people found it easier to answer “A Little” or “Somewhat” than “Not at All.”

With regard to Person parameters in class 2 the trend has moved slightly to the right, but the category frequencies are more or less equally distributed for all items (in a *normal distribution*) (see Figure 1). The raw-score mean was 17.082 (SD = 2.91). Examination of the item-fitting in class two showed an acceptable fit of the items to the data (*Q* = 0.14–0.28).

What follows is the output for class 3, expected to include about 20% of the participants. In class 3, with size 0.19398, the threshold’s trend is quite different from what appeared in the other classes, and it ranges from −2.771 to 1.458 (see Table 5).

With regard to Person parameters, the distribution seems to be a uniform distribution, in which values have the same probability of occurrence (rectangular distribution) (see Figure 1). The raw-score mean is 16.522 (SD = 5.84). The examination of the item-fitting in class 3 showed a satisfactory fit of the items to the data (*Q* = 0.12–0.18).

### Item response theory analysis: Multilog

Inspection residuals related with model-data fit indicates that the majority of residuals were not higher than 0.05. This indicates a satisfactory fit to the GRM model to our data. Table 6 illustrates the discrimination and threshold parameters associated with each item of the IOS. All of the items reported satisfactory discrimination values, with the exception of item 4 (“I am aware of my inner thoughts about things”), which seems to have a very high discrimination (see Table 6).

**TABLE 6:** IRT parameter estimates and standard errors for the IOS – Grade Response Model.

Item	α	β*1*	β*2*	β*3*	β*4*
1) I am aware of the things I am doing	2.01 (0.10)	−3.55 (0.28)	−1.94 (0.10)	0.05 (0.04)	1.50 (0.07)
2) I am able to solve difficult problems	1.00 (0.00)	−1.39 (0.00)	−0.41 (0.00)	0.41 (0.00)	1.39 (0.00)
3) I am often surprised about connections that I am able to make between my thoughts and my feelings	2.69 (0.13)	−2.76 (0.15)	−1.44 (0.06)	0.16 (0.04)	1.46 (0.06)
4) I am aware of my inner thoughts about things	4.97 (0.00)	−1.39 (0.00)	−0.41 (0.00)	0.41 (0.00)	−7.30 (0.00)
5) I am in tune with my feelings	0.90 (0.07)	−2.34 (0.19)	−0.44 (0.09)	1.48 (0.13)	3.49 (0.25)
6) I can change my behavior when I realize that things are not going well	1.00 (0.00)	−1.39 (0.00)	−0.41 (0.00)	0.41 (0.00)	1.39 (0.00)
7) I am able to be reflective about myself	0.81 (0.07)	−2.62 (0.25)	−0.91 (0.12)	1.46 (0.15)	3.51 (0.31)

α, discrimination parameter; β1, β2, β3, β4, threshold parameters.

Table 6 also shows values of threshold parameters for each item (β1 to β4) of the IOS. Values for the items 2 and 6 were lower, indicating that individuals with low levels of the latent trait were still endorsing the item (in the keyed direction), and higher for the items 1,3,5, and 7 (see Table 6).

**FIGURE 2:**
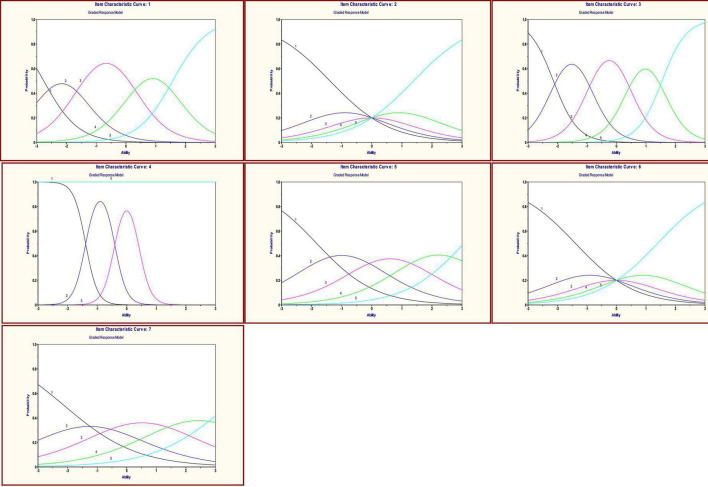
Item Characteristic Curves of the seven items of the IOS – Grade Response Model. Category response curves for the seven items of the IOS. From left to right in the first column (item 1, item 2, item 3); From left to right in the second column (item 4, item 5, item 6); in the third column (item 7).

As reported in Figure 2, the Item Characteristic Curves of the items show that item 1 and item 3 have high discrimination and non-overlapping response option categories, while item 4 shows a large overlap between option categories (see Figure 2). Moreover, item 2 and item 6 seem to perform poorly.

**FIGURE 3:**
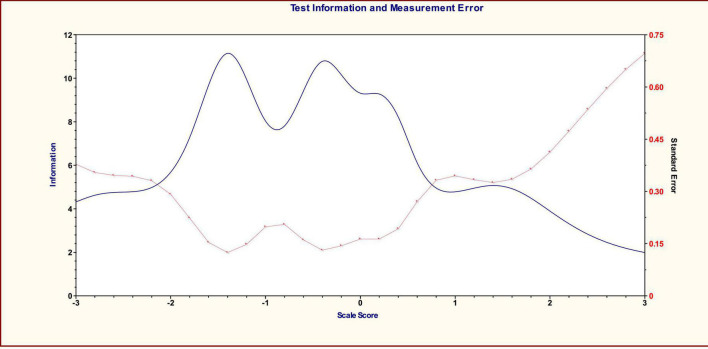
Test Information Function and Measurement Error Curves – Grade Response Model. The test information curve is represented by the solid line. The standard error of measurement curve is represented by the dotted line.

Figure 3 shows the Test Information Function (TIF) for the IOS (see Figure 3). The TIF values are relatively high across the portion of the range of the latent trait between the values of −2.15 and 0.75 (scale scores), with a decrease for those people higher than 1 *SD* unit above the mean. From 1 *SD* unit above the mean to 2 *SD* units under the mean lies the interval where the standard error curve shows its minimum values (Figure 3).

## Discussion

Insight theory represents an empirically grounded framework for the understanding of the individual’s functioning, with increasing applications in several psychological domains (e.g., Gori et al., 2021b,^2022^). In this view, the use of short scales, agile in administration and scoring, jargon free, and psychometrically solid appears to be of great utility for assessing and monitoring insight orientation in several sectors and contexts. Indeed, the development of psychometrically-sound short measures can be useful not only in clinical activity to monitor the outcomes of the psychotherapy process (Castonguay and Hill, 2007) but it is also encouraged by Work and Organization Psychology scholars because it allows researchers to decrease costs of research and intervention but maintain high reliability during measurement (Whiston, 1996, 2001).

With this in mind, the aim of this study was to examine and deeply verify the psychometrics characteristics of the Insight Orientation Scale (IOS; Gori et al., 2015) by applying Item Response Theory (IRT), and using two different software (WINMIRA and Multilog). After choosing the best model (PCM) based on likelihood statistics (Consistent Akaike’s Information Criterion – CAIC) (Bozdogan, 1987), we observed a three-class solution as the best fitting solution for our data. The proportions of subjects who were classified into the three classes were LC1 = 41.23%, LC2 = 39.37%, and LC3 = 19.4%. In LC1 and LC2 the trends for the thresholds were quite similar. In LC3 the threshold’s range is narrowed and the threshold parameters of item 3, item 4, item 5 and item 7, have arranged themselves in a different way. The frequencies of the raw scores of subjects were more or less equally distributed in LC1 and LC2. Most of subjects in LC2 tended to have high scores; the raw-score mean in this class was 17.082 (SD = 2.91). The second best performance was in LC3; subjects obtained a raw-score mean of 16.522 (SD = 5.84). In LC1, subjects obtained the lowest mean score: 13.034 (SD = 2.87). In each of the three classes the most “difficult” items were item 3 and item 4, on the basis of the item location parameters of each class; thus, these were the same items with the highest values of discrimination (α = 2.69 and α = 4.97). Instead, subjects found it “easier” to respond in the direction of endorsing insight to items 1 and 7 in LC1 (Item Location = −0.51183, −0.67651, respectively); to items 1 and 2 in LC2 (Item Location = −0.81921, −1.10372, respectively); and to items 1 and 7 in LC3 (Item Location = −0.71154, −0.39799, respectively). Differences among the three classes seem not to be related to people’s gender or age. Concerning item discrimination, most of the items showed reasonable discrimination parameters. Some of the items did have low threshold parameters, in particular item 2 and item 6, suggesting that these are relatively “easy” to endorse. With regard to the ICCs plots we observed that the items 2 and 6 have a substantial overlap among the response options, and this may be indicative of a redundancy in response options for these items. Nonetheless, the test information value shows that the instrument as a whole has satisfactory measurement precision across most of the latent trait range.

This study has some limitations to be highlighted and discussed. First, the IOS requires self-assessing a dimension on which respondents may not have full awareness, even if the insight orientation analysis was not envisaged with a single direct question but with different items that evaluate some core aspects. Furthermore, the use of self-report scales exposes to numerous known biases, such as that of social desirability. Although the accuracy and advantages of self-report measures have often been reported in different areas (e.g., Hudson et al., 2020), the use of a multi-method approach could be an important challenge for future research to overcome these issues inherent in the type of measurement instrument. Furthermore, since our participants were Italian subjects, thus the generalizability of the results in different cultures should take place with caution. Thus, future research should confirm these results in participants of different geographical areas and explore differences related to the culture and living contest.

## Conclusion

The IRT analysis showed that the three-class solution had the best-fitting solution for our data. Most of the items showed reasonable discrimination parameters, suggesting that they are able at discriminating participants across the different range of the latent trait. In Class 1 (LC1 = 41.23%) and Class 2 (LC2 = 39.37%) the thresholds followed a homogeneous trend and the raw scores of subjects were more or less equally distributed in these two classes. This means that for the majority of the participants (about 80% of subjects) every response category is representative for an interval of the individual parameter dimension.

The comparison between the two methods for conducting IRT analyses underlined that the most “difficult” items in each class (item 3 = “I am often surprised about connections that I am able to make between my thoughts and my feelings” and item 4 = “I am aware of my inner thoughts about things”) are the items with the item-total correlations (item 3 = 0.43; and item 4 = 0.47) and with the highest values of discrimination (α = 2.69 and α = 4.97); in contrast, item 2 (“I am able to solve difficult problems”) showed the largest item-total correlations (item 2 = 0.70) with low threshold parameters and substantial overlap between the response categories.

These results allow an in-depth study of the psychometric properties of the IOS at such a promising scale not only in clinical domains but also in the Work and Organizational Psychology framework. Through a comparison between two methods (CRT, used in the paper of Gori et al., 2015, and the IRT, explored in this study) and the statistical goodness of the scale, which given its properties of brevity and agility, the IOS appears a scale that could be usefully used for assessing insight orientation in both research and practice. This approach could be of great value in organizational contexts, particularly from a positive healthy organization perspective (Di Fabio, 2017). Positive healthy organizations focus on encouraging and developing positive proactive behaviors, aimed at balancing the relationship between job demands and employees’ psychological resources and strength, also advancing positive working environments connotated by workplace relational civility (Di Fabio and Gori, 2016b), acceptance of change (Di Fabio and Gori, 2016a) and sustainable innovative organizational behaviors (Duradoni and Di Fabio, 2019) to respond to the challenges of the XXI century. Previous results showed that insight orientation meditates the associations between personality traits and a prominent variable involved in maintaining the balance between job demands and job resources: job crafting (Gori et al., 2021b). Thus, the enhancement of insight orientation can lead workers to the achievement of new awareness about cognitive, emotional and social aspects relevant to facilitate job crafting with the aim of also reducing workplace stress (Ramaci et al., 2019; Caponnetto et al., 2022; Torvisco et al., 2022). Furthermore, job crafting emerged in the literature as a positive variable associated with a large array of well-being and positive organizational outcomes also in vulnerable workers (Svicher and Di Fabio, 2021). On this basis, insight orientation could be a new arrow to be launched also in organizational contexts to increase not only well-being but also decent work (Di Fabio and Kenny, 2019; Zammitti et al., 2021; Svicher et al., 2022a,b) as reference coordinates for sustainable development.

## Data availability statement

The raw data supporting the conclusions of this article will be made available by the authors, without undue reservation.

## Ethics statement

The studies involving human participants were reviewed and approved by the Ethics Committee of Integrated Psychodynamic Psychotherapy Institute (IPPI). The patients/participants provided their written informed consent to participate in this study.

## Author contributions

AG: conceptualization, methodology, and formal analysis. AG and ET: investigation, data curation, and writing—original draft preparation. AG, ET, DS, AS, and AD: writing—review and editing. AG, DS, and AD: supervision. All authors have read and agreed to the published version of the manuscript.
